# Bis{*S*-benzyl 3-[(phen­yl)(pyridin-2-yl)methyl­idene]dithio­carbazato}zinc acetonitrile monosolvate

**DOI:** 10.1107/S1600536812009592

**Published:** 2012-03-10

**Authors:** Thahira B. S. A. Ravoof, Siti Aminah Omar, Mohamed Ibrahim Mohamed Tahir, Karen A. Crouse

**Affiliations:** a Department of Chemistry, Faculty of Science, Universiti Putra Malaysia, 43400 UPM, Serdang, Selangor, Malaysia

## Abstract

In the title compound, [Zn(C_20_H_16_N_3_S_2_)_2_]·CH_3_CN, two different Schiff base moieties coordinate to the central Zn^II^ ion as tridentate *N*,*N*′,*S*-chelating ligands, creating a distorted octa­hedral environment [the smallest angle being 73.24 (6)° and the widest angle being 155.73 (7)°], with the two S atoms in *cis* positions. The dihedral angle between the mean planes of the two coordinating ligands is 83.65 (5)°. The crystal packing is consolidated by weak C—H⋯N hydrogen-bonding inter­actions.

## Related literature

For background to the coordination chemistry of hydrazine carbodithio­ates, see: Ravoof *et al.* (2010 ▶). For the synthesis, see: Ravoof *et al.* (2004 ▶). For related structures, see: Hossain *et al.* (1996 ▶); Paulus *et al.* (2011 ▶). For H-atom treatment in the refinement, see: Cooper *et al.* (2010 ▶).
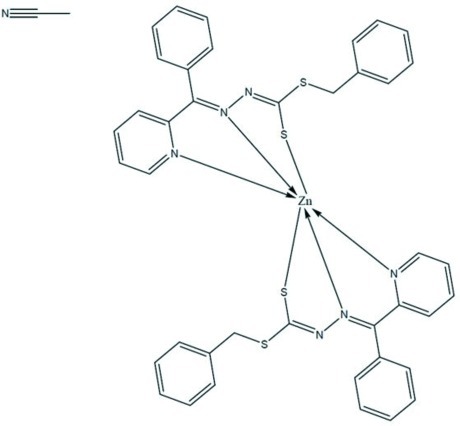


## Experimental

### 

#### Crystal data

[Zn(C_20_H_16_N_3_S_2_)_2_]·C_2_H_3_N
*M* *_r_* = 831.43
Monoclinic, 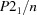

*a* = 12.5918 (3) Å
*b* = 14.0025 (3) Å
*c* = 22.2129 (5) Å
β = 100.429 (2)°
*V* = 3851.79 (14) Å^3^
*Z* = 4
Mo *K*α radiation
μ = 0.90 mm^−1^
*T* = 150 K
0.27 × 0.18 × 0.04 mm

#### Data collection

Oxford Diffraction Gemini CCD diffractometer
Absorption correction: multi-scan (*CrysAlis PRO*; Agilent, 2011 ▶) *T*_min_ = 0.85, *T*_max_ = 0.96
17196 measured reflections
8678 independent reflections
7022 reflections with *I* > 2.0σ(*I*)
*R*_int_ = 0.032

#### Refinement

*R*[*F* ^2^ > 2σ(*F* ^2^)] = 0.038
*wR*(*F* ^2^) = 0.081
*S* = 0.99
8677 reflections
487 parameters
H-atom parameters constrained
Δρ_max_ = 0.63 e Å^−3^
Δρ_min_ = −0.56 e Å^−3^

### 

Data collection: *CrysAlis CCD* (Oxford Diffraction, 2006 ▶); cell refinement: *CrysAlis PRO* (Agilent, 2011 ▶); data reduction: *CrysAlis PRO*; program(s) used to solve structure: *SIR92* (Altomare *et al.*, 1994 ▶); program(s) used to refine structure: *CRYSTALS* (Betteridge *et al.*, 2003 ▶); molecular graphics: *CAMERON* (Watkin *et al.*, 1996 ▶); software used to prepare material for publication: *CRYSTALS*.

## Supplementary Material

Crystal structure: contains datablock(s) global, I. DOI: 10.1107/S1600536812009592/wm2598sup1.cif

Structure factors: contains datablock(s) I. DOI: 10.1107/S1600536812009592/wm2598Isup2.hkl

Additional supplementary materials:  crystallographic information; 3D view; checkCIF report

## Figures and Tables

**Table 1:** Selected bond lengths (Å)

Zn1—N102	2.1346 (17)
Zn1—S105	2.4403 (6)
Zn1—N115	2.2288 (17)
Zn1—N202	2.1160 (17)
Zn1—S205	2.4516 (6)
Zn1—N215	2.3188 (17)

**Table 2:** Hydrogen-bond geometry (Å, °)

*D*—H⋯*A*	*D*—H	H⋯*A*	*D*⋯*A*	*D*—H⋯*A*
C216—H2161⋯N103	0.95	2.62	3.285 (3)	127

**Fig. 1.**
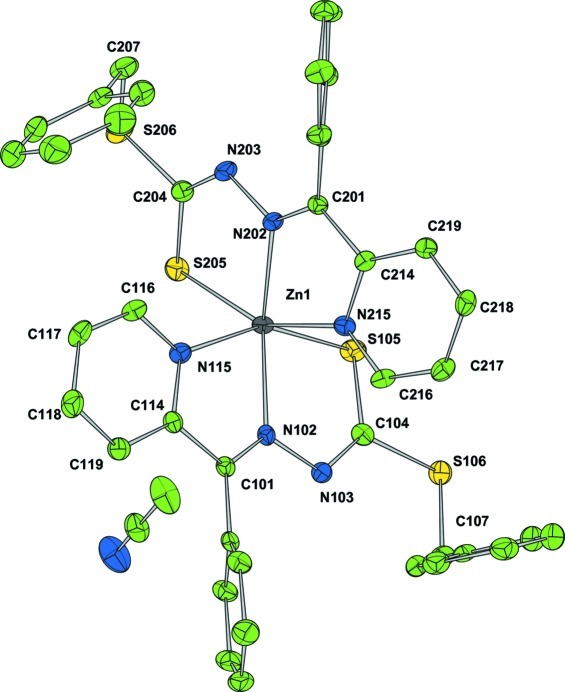
The title compound with displacement ellipsoids drawn at the 50% probability level. H atoms are shown as spheres of arbitary radius.

**Fig. 2.**
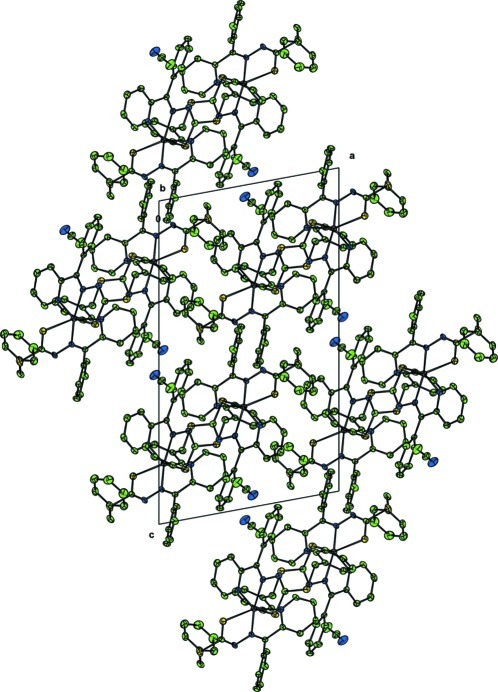
Molecular packing diagram of the title compound viewed along the b axis. Hydrogen atoms are omitted for clarity.

## supplementary crystallographic information

### Comment

The title compound was preferentially formed during the synthesis of the tridentate Schiff base with zinc saccharinate, by eliminating the saccharinate anion and instead coordinating one metal ion with two tridentate deprotonated Schiff base moieties. Background on the coordination chemistry of hydrazine carbodithioates is given by Ravoof *et al.* (2010). Similar Cu(II) complexes have been previously synthesized by Hossain *et al.* (1996).

There is one independent molecule in the asymmetric unit which contains the Zn^II^ ion coordinated to two tridentate Schiff bases *via* the pyridyl nitrogen (N115, N215), azomethine nitrogen (N102, N202) and thiolate sulfur (S105, S205) atoms (Fig. 1). A solvent acetonitrile molecule in also present in the lattice. The coordination of the metal ion is distorted octahedral with equatorial angles ranging from 73.24 (6)° to 120.54 (5)°. The angle between the planes containing the atoms of the tridentate chelating rings is 83.65 (5). The planes containing the benzyl rings attached to the sulfur atom on both Schiff bases are almost parallel to each other with an angle of 16.41 (10). Similarly, the benzyl rings on the ketone moiety of the two Schiff bases are also almost parallel with an angle of 11.69 (9)°. However, the pyridine rings of the ketone moiety of the two Schiff bases are at an angle of 66.52 (10)°.

The packing diagram viewed along the *b* axis shows an arrangement where the benzyl ring of the ketone moiety of Schiff base 2 are arranged in such a way that it is facing each other between molecules. The crystal packing is consolidated by weak C—H···N hydrogen bonding interactions (Table 2).

For related structures, see: Hossain *et al.* (1996); Ravoof *et al.* (2010); Paulus *et al.* (2011).

### Experimental

Zinc saccharinate, [Zn(sac)_2_(H_2_O)_4_]. 2H_2_O was prepared according to the method outlined in Ravoof *et al.* (2004). The 2-benzoylpyridine Schiff base of *S*-benzyldithiocarbazate was prepared following the method by Hossain *et al.* (1996). The Schiff base was dissolved in acetonitrile (50 ml) and mixed with an equimolar quantity of zinc saccharinate in acetonitrile (25 ml). The resulting mixture was heated on a water bath until the volume reduced to about 30 ml. On standing overnight, the mixture yielded orange crystals which were filtered off, washed with acetonitrile and dried in a desiccator over anhydrous silica gel, overnight. Crystals of the zinc complex suitable for X-ray diffraction analysis were obtained by recrystallisation from a mixture of acetonitrile, THF and chloroform. Slow evaporation over 3 weeks yielded crystals suitable for diffraction experiments.

### Refinement

H atoms were all located in difference maps, but those attached to C atoms were repositioned geometrically. The H atoms were initially refined with soft restraints on the bond lengths and angles to regularize their geometry (C—H in the range 0.93–0.98, N—H in the range 0.86–0.89 Å) and *U*_iso_(H) (in the range 1.2–1.5 times *U*_eq_ of the parent atom), after which the positions were refined with riding constraints (Cooper *et al.*, 2010).

### Crystal data

**Table:**

[Zn(C_20_H_16_N_3_S_2_)_2_]·C_2_H_3_N	*F*(000) = 1720
*M**_r_* = 831.43	*D*_x_ = 1.434 Mg m^−^^3^
Monoclinic, *P*2_1_/*n*	Mo *K*α radiation, λ = 0.71073 Å
Hall symbol: -P 2yn	Cell parameters from 6190 reflections
*a* = 12.5918 (3) Å	θ = 2–29°
*b* = 14.0025 (3) Å	µ = 0.90 mm^−^^1^
*c* = 22.2129 (5) Å	*T* = 150 K
β = 100.429 (2)°	Prismatic, yellow
*V* = 3851.79 (14) Å^3^	0.27 × 0.18 × 0.04 mm
*Z* = 4	

### Data collection

**Table:**

Oxford Diffraction Gemini CCD diffractometer	8678 independent reflections
Radiation source: sealed X-ray tube, Oxford Diffraction Enhance X-ray	7022 reflections with *I* > 2.0σ(*I*)
Graphite monochromator	*R*_int_ = 0.032
φ scans	θ_max_ = 28.8°, θ_min_ = 2.2°
Absorption correction: multi-scan (*CrysAlis PRO*; Agilent, 2011)	*h* = −12→17
*T*_min_ = 0.85, *T*_max_ = 0.96	*k* = −17→17
17196 measured reflections	*l* = −28→30

### Refinement

**Table:**

Refinement on *F*^2^	Primary atom site location: structure-invariant direct methods
Least-squares matrix: full	Hydrogen site location: difference Fourier map
*R*[*F*^2^ > 2σ(*F*^2^)] = 0.038	H-atom parameters constrained
*wR*(*F*^2^) = 0.081	Method = Modified Sheldrick *w* = 1/[σ^2^(*F*^2^) + ( 0.03*P*)^2^ + 2.81*P*], where *P* = (max(*F*_o_^2^,0) + 2*F*_c_^2^)/3
*S* = 0.99	(Δ/σ)_max_ = 0.001
8677 reflections	Δρ_max_ = 0.63 e Å^−^^3^
487 parameters	Δρ_min_ = −0.56 e Å^−^^3^
0 restraints	

### Special details

**Table:**

Experimental. The crystal was placed in the cold stream of an Oxford Cryosystems open-flow nitrogen cryostat (Cosier & Glazer, 1986) with a nominal stability of 0.1 K.Cosier, J. & Glazer, A.*M*., 1986. J. Appl. Cryst. 105–107.

### Fractional atomic coordinates and isotropic or equivalent isotropic displacement parameters (Å^2^)

**Table:**

	*x*	*y*	*z*	*U*_iso_*/*U*_eq_	
Zn1	0.52827 (2)	0.992001 (17)	0.315553 (11)	0.0157	
C101	0.54238 (16)	0.96909 (14)	0.18279 (9)	0.0151	
N102	0.57756 (13)	1.02145 (12)	0.23042 (8)	0.0145	
N103	0.66583 (14)	1.07951 (12)	0.22781 (8)	0.0165	
C104	0.69996 (17)	1.12459 (14)	0.27968 (9)	0.0170	
S105	0.64556 (5)	1.12917 (4)	0.34537 (3)	0.0223	
S106	0.82077 (5)	1.18896 (4)	0.28549 (3)	0.0218	
C107	0.86887 (19)	1.16235 (16)	0.21458 (10)	0.0227	
C108	0.92336 (18)	1.06688 (16)	0.21381 (10)	0.0201	
C109	1.02461 (19)	1.04967 (18)	0.24911 (11)	0.0271	
C110	1.0748 (2)	0.96228 (19)	0.24752 (11)	0.0306	
C111	1.0238 (2)	0.88970 (18)	0.21068 (11)	0.0276	
C112	0.92326 (19)	0.90545 (17)	0.17534 (10)	0.0257	
C113	0.87376 (18)	0.99394 (16)	0.17643 (10)	0.0212	
C114	0.45800 (16)	0.89875 (15)	0.19093 (9)	0.0160	
N115	0.43995 (14)	0.88792 (12)	0.24871 (8)	0.0173	
C116	0.36456 (17)	0.82562 (16)	0.25873 (10)	0.0211	
C117	0.30530 (18)	0.77079 (16)	0.21274 (11)	0.0247	
C118	0.32364 (19)	0.78172 (17)	0.15367 (11)	0.0264	
C119	0.40036 (18)	0.84708 (16)	0.14234 (10)	0.0220	
C120	0.58251 (17)	0.97419 (15)	0.12405 (9)	0.0169	
C121	0.62547 (18)	0.89391 (16)	0.10049 (10)	0.0218	
C122	0.6655 (2)	0.90042 (18)	0.04628 (11)	0.0288	
C123	0.6611 (2)	0.98605 (18)	0.01499 (10)	0.0289	
C124	0.6161 (2)	1.06568 (18)	0.03757 (10)	0.0281	
C125	0.57791 (19)	1.06006 (16)	0.09211 (10)	0.0223	
C201	0.59004 (16)	0.84982 (14)	0.41713 (9)	0.0150	
N202	0.51528 (13)	0.90998 (12)	0.39390 (8)	0.0157	
N203	0.42040 (14)	0.90721 (13)	0.41716 (8)	0.0193	
C204	0.34919 (17)	0.96989 (16)	0.39193 (10)	0.0189	
S205	0.35673 (4)	1.05422 (4)	0.33664 (3)	0.0213	
S206	0.22568 (5)	0.96742 (5)	0.41839 (3)	0.0309	
C207	0.2312 (2)	0.85102 (19)	0.45623 (11)	0.0322	
C208	0.22822 (18)	0.76718 (18)	0.41302 (11)	0.0276	
C209	0.14642 (19)	0.7582 (2)	0.36166 (12)	0.0346	
C210	0.1418 (2)	0.6803 (2)	0.32314 (12)	0.0394	
C211	0.2202 (2)	0.6104 (2)	0.33423 (13)	0.0409	
C212	0.3031 (2)	0.61924 (19)	0.38429 (13)	0.0391	
C213	0.3062 (2)	0.69618 (18)	0.42340 (11)	0.0310	
C214	0.68809 (17)	0.85023 (14)	0.38898 (9)	0.0155	
N215	0.67706 (14)	0.89154 (12)	0.33326 (8)	0.0163	
C216	0.76099 (17)	0.88769 (15)	0.30420 (10)	0.0192	
C217	0.85827 (18)	0.84506 (16)	0.32896 (10)	0.0220	
C218	0.87086 (17)	0.80787 (15)	0.38733 (10)	0.0208	
C219	0.78445 (17)	0.81014 (15)	0.41769 (10)	0.0192	
C220	0.58100 (16)	0.77931 (15)	0.46577 (9)	0.0157	
C221	0.59856 (18)	0.68281 (16)	0.45448 (10)	0.0204	
C222	0.58656 (19)	0.61414 (16)	0.49780 (11)	0.0245	
C223	0.55935 (19)	0.64118 (17)	0.55269 (10)	0.0260	
C224	0.54377 (18)	0.73704 (17)	0.56474 (10)	0.0237	
C225	0.55425 (17)	0.80586 (16)	0.52144 (9)	0.0189	
C301	0.5638 (2)	0.54258 (19)	0.08460 (13)	0.0421	
C302	0.5150 (2)	0.63022 (19)	0.05801 (11)	0.0308	
N303	0.4777 (2)	0.69814 (17)	0.03609 (10)	0.0421	
H1071	0.9213	1.2132	0.2111	0.0279*	
H1072	0.8069	1.1671	0.1809	0.0280*	
H1091	1.0597	1.0995	0.2750	0.0331*	
H1101	1.1443	0.9511	0.2701	0.0389*	
H1111	1.0583	0.8288	0.2094	0.0346*	
H1121	0.8877	0.8560	0.1500	0.0322*	
H1131	0.8041	1.0054	0.1510	0.0267*	
H1161	0.3528	0.8199	0.3002	0.0264*	
H1171	0.2530	0.7272	0.2224	0.0297*	
H1181	0.2835	0.7452	0.1212	0.0328*	
H1191	0.4138	0.8567	0.1019	0.0273*	
H1211	0.6288	0.8342	0.1222	0.0266*	
H1221	0.6965	0.8447	0.0307	0.0360*	
H1231	0.6890	0.9897	−0.0220	0.0349*	
H1241	0.6106	1.1255	0.0160	0.0344*	
H1251	0.5479	1.1150	0.1079	0.0278*	
H2071	0.2968	0.8479	0.4886	0.0407*	
H2072	0.1655	0.8490	0.4758	0.0406*	
H2091	0.0920	0.8079	0.3527	0.0422*	
H2101	0.0845	0.6752	0.2897	0.0485*	
H2111	0.2179	0.5566	0.3074	0.0489*	
H2121	0.3592	0.5728	0.3916	0.0497*	
H2131	0.3634	0.7018	0.4592	0.0396*	
H2161	0.7536	0.9149	0.2645	0.0237*	
H2171	0.9146	0.8431	0.3063	0.0266*	
H2181	0.9384	0.7810	0.4067	0.0256*	
H2191	0.7890	0.7845	0.4586	0.0233*	
H2211	0.6186	0.6641	0.4165	0.0255*	
H2221	0.5970	0.5479	0.4891	0.0300*	
H2231	0.5512	0.5940	0.5829	0.0323*	
H2241	0.5264	0.7553	0.6038	0.0282*	
H2251	0.5427	0.8719	0.5299	0.0240*	
H3012	0.5394	0.5297	0.1234	0.0643*	
H3011	0.6406	0.5507	0.0906	0.0637*	
H3013	0.5432	0.4920	0.0566	0.0642*	

### Atomic displacement parameters (Å^2^)

**Table:**

	*U*^11^	*U*^22^	*U*^33^	*U*^12^	*U*^13^	*U*^23^
Zn1	0.01798 (13)	0.01668 (12)	0.01312 (12)	0.00072 (10)	0.00433 (10)	0.00122 (10)
C101	0.0149 (10)	0.0139 (10)	0.0156 (10)	0.0010 (8)	0.0004 (8)	0.0023 (8)
N102	0.0142 (8)	0.0126 (8)	0.0155 (8)	−0.0019 (7)	−0.0003 (7)	0.0011 (7)
N103	0.0180 (9)	0.0146 (9)	0.0167 (9)	−0.0030 (7)	0.0022 (7)	0.0005 (7)
C104	0.0204 (11)	0.0111 (10)	0.0189 (11)	0.0026 (8)	0.0023 (9)	0.0022 (8)
S105	0.0278 (3)	0.0216 (3)	0.0190 (3)	−0.0035 (2)	0.0079 (2)	−0.0067 (2)
S106	0.0242 (3)	0.0198 (3)	0.0209 (3)	−0.0079 (2)	0.0026 (2)	−0.0038 (2)
C107	0.0239 (12)	0.0229 (12)	0.0221 (11)	−0.0070 (10)	0.0060 (10)	0.0035 (9)
C108	0.0207 (11)	0.0233 (12)	0.0178 (11)	−0.0053 (9)	0.0077 (9)	0.0037 (9)
C109	0.0259 (12)	0.0316 (13)	0.0232 (12)	−0.0078 (11)	0.0029 (10)	−0.0008 (10)
C110	0.0218 (12)	0.0416 (15)	0.0280 (13)	0.0016 (11)	0.0032 (11)	0.0045 (11)
C111	0.0287 (13)	0.0325 (14)	0.0241 (12)	0.0064 (11)	0.0113 (11)	0.0029 (10)
C112	0.0284 (13)	0.0291 (13)	0.0215 (12)	−0.0023 (11)	0.0101 (10)	−0.0046 (10)
C113	0.0178 (11)	0.0282 (12)	0.0181 (11)	−0.0031 (9)	0.0044 (9)	−0.0012 (9)
C114	0.0156 (10)	0.0150 (10)	0.0166 (10)	0.0012 (8)	0.0009 (9)	−0.0003 (8)
N115	0.0180 (9)	0.0168 (9)	0.0178 (9)	−0.0003 (7)	0.0050 (8)	−0.0008 (7)
C116	0.0220 (11)	0.0205 (11)	0.0227 (11)	−0.0017 (9)	0.0098 (10)	−0.0003 (9)
C117	0.0195 (11)	0.0230 (12)	0.0333 (13)	−0.0069 (10)	0.0091 (10)	−0.0027 (10)
C118	0.0226 (12)	0.0273 (12)	0.0283 (13)	−0.0085 (10)	0.0019 (11)	−0.0059 (10)
C119	0.0224 (11)	0.0243 (12)	0.0190 (11)	−0.0057 (10)	0.0031 (10)	−0.0012 (9)
C120	0.0163 (10)	0.0204 (11)	0.0128 (10)	−0.0057 (9)	−0.0007 (8)	−0.0006 (8)
C121	0.0255 (12)	0.0211 (11)	0.0185 (11)	−0.0004 (9)	0.0031 (10)	−0.0003 (9)
C122	0.0303 (13)	0.0330 (14)	0.0243 (12)	−0.0030 (11)	0.0082 (11)	−0.0088 (11)
C123	0.0296 (13)	0.0420 (15)	0.0164 (11)	−0.0131 (12)	0.0077 (10)	−0.0047 (11)
C124	0.0373 (14)	0.0281 (13)	0.0182 (11)	−0.0119 (11)	0.0035 (11)	0.0046 (10)
C125	0.0290 (12)	0.0214 (11)	0.0156 (11)	−0.0047 (10)	0.0021 (10)	−0.0004 (9)
C201	0.0161 (10)	0.0152 (10)	0.0137 (10)	−0.0003 (8)	0.0028 (9)	−0.0009 (8)
N202	0.0144 (8)	0.0188 (9)	0.0151 (9)	0.0016 (7)	0.0056 (7)	−0.0001 (7)
N203	0.0153 (9)	0.0253 (10)	0.0192 (9)	0.0040 (8)	0.0083 (8)	0.0017 (8)
C204	0.0176 (10)	0.0238 (11)	0.0164 (10)	0.0037 (9)	0.0058 (9)	−0.0041 (9)
S205	0.0210 (3)	0.0203 (3)	0.0225 (3)	0.0064 (2)	0.0035 (2)	0.0022 (2)
S206	0.0202 (3)	0.0392 (4)	0.0365 (3)	0.0097 (3)	0.0140 (3)	0.0043 (3)
C207	0.0254 (13)	0.0447 (16)	0.0299 (13)	0.0013 (12)	0.0136 (11)	0.0072 (12)
C208	0.0206 (12)	0.0377 (14)	0.0267 (12)	−0.0046 (11)	0.0101 (10)	0.0079 (11)
C209	0.0185 (12)	0.0499 (17)	0.0352 (14)	−0.0056 (12)	0.0048 (11)	0.0121 (13)
C210	0.0319 (15)	0.0522 (18)	0.0321 (14)	−0.0204 (14)	0.0006 (12)	0.0077 (13)
C211	0.0484 (17)	0.0385 (16)	0.0355 (15)	−0.0211 (14)	0.0063 (14)	0.0019 (12)
C212	0.0406 (16)	0.0307 (14)	0.0448 (16)	−0.0017 (12)	0.0051 (14)	0.0070 (12)
C213	0.0270 (13)	0.0353 (14)	0.0298 (13)	−0.0052 (11)	0.0028 (11)	0.0077 (11)
C214	0.0160 (10)	0.0133 (10)	0.0177 (10)	0.0007 (8)	0.0047 (9)	−0.0003 (8)
N215	0.0184 (9)	0.0145 (9)	0.0159 (9)	0.0017 (7)	0.0029 (8)	0.0022 (7)
C216	0.0216 (11)	0.0183 (11)	0.0200 (11)	0.0002 (9)	0.0098 (9)	0.0024 (9)
C217	0.0183 (11)	0.0235 (12)	0.0263 (12)	0.0019 (9)	0.0094 (10)	0.0000 (10)
C218	0.0146 (10)	0.0190 (11)	0.0286 (12)	0.0032 (9)	0.0033 (10)	0.0020 (9)
C219	0.0193 (11)	0.0200 (11)	0.0184 (11)	−0.0004 (9)	0.0037 (9)	0.0031 (9)
C220	0.0119 (10)	0.0192 (10)	0.0161 (10)	0.0022 (8)	0.0027 (8)	0.0030 (8)
C221	0.0215 (11)	0.0228 (11)	0.0187 (11)	0.0015 (9)	0.0085 (10)	−0.0004 (9)
C222	0.0295 (13)	0.0168 (11)	0.0295 (12)	0.0035 (10)	0.0114 (11)	0.0027 (9)
C223	0.0283 (12)	0.0268 (12)	0.0245 (12)	0.0029 (10)	0.0089 (11)	0.0099 (10)
C224	0.0274 (12)	0.0286 (12)	0.0166 (11)	0.0044 (10)	0.0083 (10)	0.0022 (9)
C225	0.0184 (11)	0.0206 (11)	0.0176 (11)	0.0024 (9)	0.0030 (9)	0.0000 (9)
C301	0.0438 (17)	0.0337 (15)	0.0425 (16)	0.0025 (13)	−0.0089 (14)	−0.0034 (13)
C302	0.0297 (13)	0.0346 (14)	0.0253 (13)	−0.0064 (12)	−0.0024 (11)	−0.0048 (11)
N303	0.0498 (15)	0.0334 (13)	0.0372 (13)	−0.0009 (11)	−0.0082 (12)	−0.0027 (11)

### Geometric parameters (Å, º)

**Table:**

Zn1—N102	2.1346 (17)	C201—N202	1.299 (3)
Zn1—S105	2.4403 (6)	C201—C214	1.481 (3)
Zn1—N115	2.2288 (17)	C201—C220	1.483 (3)
Zn1—N202	2.1160 (17)	N202—N203	1.385 (2)
Zn1—S205	2.4516 (6)	N203—C204	1.306 (3)
Zn1—N215	2.3188 (17)	C204—S205	1.719 (2)
C101—N102	1.297 (3)	C204—S206	1.760 (2)
C101—C114	1.484 (3)	S206—C207	1.829 (3)
C101—C120	1.484 (3)	C207—C208	1.512 (4)
N102—N103	1.387 (2)	C207—H2071	0.994
N103—C104	1.316 (3)	C207—H2072	1.002
C104—S105	1.722 (2)	C208—C209	1.397 (3)
C104—S106	1.753 (2)	C208—C213	1.387 (3)
S106—C107	1.825 (2)	C209—C210	1.382 (4)
C107—C108	1.504 (3)	C209—H2091	0.971
C107—H1071	0.984	C210—C211	1.381 (4)
C107—H1072	0.982	C210—H2101	0.939
C108—C109	1.392 (3)	C211—C212	1.386 (4)
C108—C113	1.391 (3)	C211—H2111	0.957
C109—C110	1.380 (4)	C212—C213	1.380 (4)
C109—H1091	0.960	C212—H2121	0.952
C110—C111	1.387 (3)	C213—H2131	0.974
C110—H1101	0.940	C214—N215	1.350 (3)
C111—C112	1.382 (3)	C214—C219	1.383 (3)
C111—H1111	0.960	N215—C216	1.335 (3)
C112—C113	1.389 (3)	C216—C217	1.384 (3)
C112—H1121	0.952	C216—H2161	0.949
C113—H1131	0.967	C217—C218	1.379 (3)
C114—N115	1.352 (3)	C217—H2171	0.942
C114—C119	1.390 (3)	C218—C219	1.380 (3)
N115—C116	1.337 (3)	C218—H2181	0.958
C116—C117	1.383 (3)	C219—H2191	0.970
C116—H1161	0.961	C220—C221	1.399 (3)
C117—C118	1.381 (3)	C220—C225	1.390 (3)
C117—H1171	0.950	C221—C222	1.388 (3)
C118—C119	1.386 (3)	C221—H2211	0.960
C118—H1181	0.952	C222—C223	1.378 (3)
C119—H1191	0.954	C222—H2221	0.961
C120—C121	1.390 (3)	C223—C224	1.389 (3)
C120—C125	1.392 (3)	C223—H2231	0.959
C121—C122	1.390 (3)	C224—C225	1.385 (3)
C121—H1211	0.962	C224—H2241	0.967
C122—C123	1.382 (3)	C225—H2251	0.961
C122—H1221	0.964	C301—C302	1.449 (4)
C123—C124	1.385 (3)	C301—H3012	0.983
C123—H1231	0.951	C301—H3011	0.958
C124—C125	1.384 (3)	C301—H3013	0.947
C124—H1241	0.962	C302—N303	1.131 (3)
C125—H1251	0.951		
			
N102—Zn1—S105	80.19 (5)	C120—C125—C124	120.4 (2)
N102—Zn1—N115	74.11 (6)	C120—C125—H1251	119.5
S105—Zn1—N115	154.15 (5)	C124—C125—H1251	120.0
N102—Zn1—N202	155.73 (7)	N202—C201—C214	115.42 (18)
S105—Zn1—N202	109.70 (5)	N202—C201—C220	125.14 (19)
N115—Zn1—N202	95.14 (6)	C214—C201—C220	119.33 (17)
N102—Zn1—S205	120.54 (5)	Zn1—N202—C201	121.37 (14)
S105—Zn1—S205	100.33 (2)	Zn1—N202—N203	121.48 (13)
N115—Zn1—S205	90.52 (5)	C201—N202—N203	116.47 (17)
N202—Zn1—S205	80.43 (5)	N202—N203—C204	113.11 (17)
N102—Zn1—N215	84.94 (6)	N203—C204—S205	130.23 (17)
S105—Zn1—N215	90.04 (5)	N203—C204—S206	115.02 (16)
N115—Zn1—N215	90.39 (6)	S205—C204—S206	114.75 (12)
N202—Zn1—N215	73.24 (6)	C204—S205—Zn1	93.26 (7)
S205—Zn1—N215	153.63 (5)	C204—S206—C207	102.05 (11)
N102—C101—C114	114.98 (18)	S206—C207—C208	113.92 (17)
N102—C101—C120	124.71 (19)	S206—C207—H2071	109.2
C114—C101—C120	120.29 (17)	C208—C207—H2071	110.3
Zn1—N102—C101	120.13 (14)	S206—C207—H2072	105.1
Zn1—N102—N103	120.93 (12)	C208—C207—H2072	109.0
C101—N102—N103	117.09 (17)	H2071—C207—H2072	109.1
N102—N103—C104	112.46 (17)	C207—C208—C209	121.2 (2)
N103—C104—S105	130.01 (17)	C207—C208—C213	121.0 (2)
N103—C104—S106	116.92 (16)	C209—C208—C213	117.8 (2)
S105—C104—S106	113.05 (12)	C208—C209—C210	121.2 (3)
C104—S105—Zn1	93.14 (7)	C208—C209—H2091	119.4
C104—S106—C107	104.51 (10)	C210—C209—H2091	119.4
S106—C107—C108	114.49 (15)	C209—C210—C211	120.1 (3)
S106—C107—H1071	104.6	C209—C210—H2101	119.3
C108—C107—H1071	109.2	C211—C210—H2101	120.7
S106—C107—H1072	107.6	C210—C211—C212	119.3 (3)
C108—C107—H1072	110.7	C210—C211—H2111	120.4
H1071—C107—H1072	110.1	C212—C211—H2111	120.2
C107—C108—C109	121.4 (2)	C211—C212—C213	120.4 (3)
C107—C108—C113	120.2 (2)	C211—C212—H2121	120.2
C109—C108—C113	118.4 (2)	C213—C212—H2121	119.3
C108—C109—C110	121.0 (2)	C208—C213—C212	121.1 (2)
C108—C109—H1091	119.2	C208—C213—H2131	118.3
C110—C109—H1091	119.8	C212—C213—H2131	120.6
C109—C110—C111	120.0 (2)	C201—C214—N215	115.68 (18)
C109—C110—H1101	121.3	C201—C214—C219	122.15 (19)
C111—C110—H1101	118.7	N215—C214—C219	122.17 (19)
C110—C111—C112	119.8 (2)	Zn1—N215—C214	111.17 (13)
C110—C111—H1111	120.4	Zn1—N215—C216	128.93 (14)
C112—C111—H1111	119.9	C214—N215—C216	117.82 (18)
C111—C112—C113	120.0 (2)	N215—C216—C217	123.1 (2)
C111—C112—H1121	120.3	N215—C216—H2161	118.6
C113—C112—H1121	119.7	C217—C216—H2161	118.3
C108—C113—C112	120.7 (2)	C216—C217—C218	118.6 (2)
C108—C113—H1131	119.3	C216—C217—H2171	119.8
C112—C113—H1131	120.0	C218—C217—H2171	121.5
C101—C114—N115	115.93 (18)	C217—C218—C219	118.9 (2)
C101—C114—C119	122.40 (19)	C217—C218—H2181	120.8
N115—C114—C119	121.67 (19)	C219—C218—H2181	120.3
Zn1—N115—C114	114.19 (13)	C214—C219—C218	119.2 (2)
Zn1—N115—C116	126.82 (14)	C214—C219—H2191	118.7
C114—N115—C116	118.53 (18)	C218—C219—H2191	122.1
N115—C116—C117	122.9 (2)	C201—C220—C221	118.48 (18)
N115—C116—H1161	116.8	C201—C220—C225	122.21 (19)
C117—C116—H1161	120.3	C221—C220—C225	119.30 (19)
C116—C117—C118	118.6 (2)	C220—C221—C222	120.3 (2)
C116—C117—H1171	119.5	C220—C221—H2211	119.8
C118—C117—H1171	121.8	C222—C221—H2211	119.9
C117—C118—C119	119.2 (2)	C221—C222—C223	119.9 (2)
C117—C118—H1181	120.2	C221—C222—H2221	119.6
C119—C118—H1181	120.6	C223—C222—H2221	120.5
C114—C119—C118	119.1 (2)	C222—C223—C224	120.1 (2)
C114—C119—H1191	120.1	C222—C223—H2231	120.3
C118—C119—H1191	120.9	C224—C223—H2231	119.6
C101—C120—C121	120.65 (19)	C223—C224—C225	120.3 (2)
C101—C120—C125	120.0 (2)	C223—C224—H2241	119.4
C121—C120—C125	119.3 (2)	C225—C224—H2241	120.3
C120—C121—C122	120.0 (2)	C220—C225—C224	120.1 (2)
C120—C121—H1211	119.9	C220—C225—H2251	120.1
C122—C121—H1211	120.0	C224—C225—H2251	119.8
C121—C122—C123	120.3 (2)	C302—C301—H3012	109.6
C121—C122—H1221	119.5	C302—C301—H3011	107.4
C123—C122—H1221	120.2	H3012—C301—H3011	111.6
C122—C123—C124	119.9 (2)	C302—C301—H3013	108.6
C122—C123—H1231	119.5	H3012—C301—H3013	110.3
C124—C123—H1231	120.6	H3011—C301—H3013	109.2
C123—C124—C125	120.0 (2)	C301—C302—N303	178.5 (3)
C123—C124—H1241	121.2	C301—C302—N303	178.5 (3)
C125—C124—H1241	118.8	C301—C302—N303	178.5 (3)

### Hydrogen-bond geometry (Å, º)

**Table:**

*D*—H···*A*	*D*—H	H···*A*	*D*···*A*	*D*—H···*A*
C216—H2161···N103	0.95	2.62	3.285 (3)	127
